# Respiratory effects of acute milk consumption among asthmatic and non-asthmatic children: a randomized controlled study

**DOI:** 10.1186/s12887-020-02319-y

**Published:** 2020-09-12

**Authors:** Yael Koren, Keren Armoni Domany, Guy Gut, Amir Hadanny, Shira Benor, Oren Tavor, Yakov Sivan

**Affiliations:** 1 Dana-Dwek Children’s Hospital, Tel Aviv-Yafo, Israel; 2 Pediatric Pulmonary Unit, The Edith Wolfson Medical Center, Holon, Israel; 3 Sackler Faculty of Medicine, Tel Aviv University, Tel Aviv, Israel; 4 Pediatric Pulmonary Institute, Ruth Children’s Hospital, Rambam Health, Care Campus, Haifa, Israel; 5 The Mina and Everard Goodman Faculty of Life Sciences, Bar-Ilan University, Ramat-Gan, Israel; 6 Department of Pediatric Pulmonology, Safra Children Hospital, Sheba Medical Center, Tel Hashomer, Israel

**Keywords:** milk, dairy, asthma, FeNO, spirometry, mucus

## Abstract

**Background:**

A commonly held public belief is that cow’s milk products increase mucus production and respiratory symptoms. Dietary milk elimination is often attempted despite lack of evidence. Our objective was to investigate whether a single exposure to cow’s milk is associated with respiratory symptoms and changes in pulmonary functions in asthmatic and non-asthmatic children.

**Methods:**

We conducted a prospective double blind, placebo-controlled trial on non-asthmatic and asthmatic children aged 6–18 years evaluated at a pediatric pulmonology unit.

The children were randomly challenged with cow’s milk or soy milk substitute. Symptoms, spirometry, fractional-exhaled nitric-oxide (FeNO), and pulse oximetry findings were obtained at baseline and at 30, 60, 90, and 120 min following challenge.

A two-way ANCOVA (with repeated measures when required) was used to compare the performances of all groups and subgroups over time. The outcome measures of each participant were compared to his/her own variables over time and in relation to his/her baseline values. In case of missing data points, missingness analysis was performed using Little’s missing completely at random (MCAR) test.

**Results:**

Fifty non-asthmatic children (26 assigned to the cow’s milk group and 24 to the soy substitute group), and 46 asthmatic children (22 in the cow’s milk group and 24 in the soy substitute group) were enrolled. Age, gender, and body mass index Z-score were comparable between the two groups. No changes in symptoms, spirometry, FeNO, or oxygen saturation measurements were observed following challenge in any of the participants in both groups, at any time point compared to baseline.

**Conclusions:**

A single exposure to cow’s milk is not associated with symptoms, bronchial inflammation, or bronchial constriction in both non-asthmatic and asthmatic children. Our findings do not support the strict elimination of dairy products from a child’s diet for the prevention of respiratory symptoms.

**Trial registration:**

This study was approved by the Tel Aviv Sourasky Medical Center Institutional Review Board and the Israeli Ministry of Health review board (Helsinki Committee, NIH #NCT02745899). Registered April 2016 https://clinicaltrials.gov/ct2/show/NCT02745899?cond=milk+asthma&rank=1.

## Background

There is a commonly held belief that cow’s milk increases airway mucus production. Dating back to the 12th century, Maimonides pointed out that the intake of milk can potentially exacerbate asthma [1]. A 2003–2004 survey conducted by the Israeli Center of Disease Control (ICDC) showed that 12% of children in the 7-12th grades abstained from dairy products mostly due to general health considerations, family lifestyle, or veganism [2]. In a 2015–2016 ICDC survey among 5,300 adolescents, 10–14% reported complete milk avoidance [3]. Data from Australia showed that almost 30% of the population believed that milk produces mucus [4, 5]. Balfour-Lynn recently reported that in their department, parents often claim that drinking milk increases mucus production and, therefore they omit milk from their children’s diet. Balfour-Lynn hence recommended that this myth be refuted [6].

One survey of 330 parents of children visiting an outpatient pediatric pulmonology clinic showed that 58% were convinced that there was a relationship between milk consumption and increased mucus production [7]. Indeed, elimination of milk products has become popular, mainly among parents of asthmatic children, under the assumption that avoiding dairy products will improve their children’s symptoms and reduce the occurrence of exacerbations [8, 9]. Despite this popular belief, no effect of cow’s milk on respiratory status was detected in several studies in adults [5, 10, 11]. To the best of our knowledge, no interventional study has similarly explored the effect of milk on respiratory symptoms and function in children.

Asthma is the most common chronic respiratory illness in children, reaching almost 10% of the pediatric population in the developed world, including Israel [12]. Cough and shortness of breath are the most common presentations of asthma, while airway bronchoconstriction, hyperresponsiveness and reversibility to drugs are its physiologic hallmarks [13]. These are measured by clinical assessment, spirometry, response to challenges, and reversibility following inhalation of bronchodilators. Asthma in children is usually associated with eosinophilic inflammation [14], which can be evaluated by the measurement of the fractional-exhaled nitric-oxide (FeNO), a sensitive marker of airway eosinophilic inflammation in asthma [15].

Avoiding milk and its products can have major impacts on health and development of children such as increasing the risk of atopic asthma [16] and the risk of an IgE mediated allergy to milk despite being tolerated before [17]. In most developed countries, over two-thirds of calcium consumption originates from milk products [18] and avoiding milk exposes children to the risk of low calcium levels and restricted growth. A recent study showed that each daily cup of cow’s milk was associated with 0.2 centimeters additional height compared to non-cow’s milk consumption in 3-year-old children [19]. Another study reported that consuming dairy products during childhood improved bone density in adulthood, and that their avoidance may lead to growth deceleration and osteoporosis [18]. This may be especially relevant to asthmatic children who are occasionally treated with steroids, which are associated with osteopenia and osteoporosis [20]. Furthermore, low intake of dairy is the primary reason that Americans do not meet their daily calcium needs [21]. Consequently, both the American Heart Association and the Canadian Ministry of Health recommend that children consume 2–4 milk servings per day [22, 23].

The 2015–2016 ICDC survey showed that only 22.5% of students in Israel consumed the daily recommended amount of calcium [3] despite the recommendation of the Israeli Society of Pediatrics’ guidelines that they consume at least 3 milk servings per day [24]. Our overall objective was to study the effect of a single exposure to cow’s milk on respiratory symptoms, oxygenation and pulmonary functions, specifically on spirometry and exhaled nitric-oxide. We hypothesized that a single exposure to cow’s milk does not induce any respiratory symptoms, oxygen desaturation or bronchoconstriction and does not cause acute airway inflammation in both non-asthmatic and asthmatic children.

## Methods

### Subjects

This was a prospective randomized, double-blind, placebo-controlled, parallel-group trial of asthmatic and non-asthmatic 6-to 18-year-old children. The study was conducted in the pediatric pulmonology unit of the “Dana-Dwek” Children’s Hospital at the Tel Aviv Medical Center in Israel. Asthmatic and non-asthmatic children were recruited from the pediatric pulmonology clinic in our hospital and those in the community. Children of the hospital personnel were also recruited. A pediatric pulmonologist re-confirmed the diagnosis of asthma for the asthmatic children and ruled out asthma for the controls. Study exclusion criteria were: (a) a known allergy to cow’s milk (including skin, gastrointestinal and respiratory manifestations), (b) acute or recent respiratory infection at the time of testing, (c) use of systemic steroids during the month preceding study initiation, (d) asthma exacerbation that had been treated with short-acting beta-agonists or inhaled corticosteroids within the 48 h prior to the trial, and (e) an underlying disease that could affect clinical assessment, spirometry, or FeNO measurements.

The study was approved by the Tel Aviv Medical Center and the Israeli Ministry of Health IRB (Helsinki Committee, NIH #NCT02745899). Informed written consent was received from all participants and their parents.

### Study design

The study design comprised a challenge with either cow’s milk or a soy milk substitute in asthmatic and non-asthmatic children and the assessment of their respiratory response to the challenge. According to the study protocol, all participants were requested to completely avoid all dairy products for the 24 h preceding the intervention. Participants from both the study and control groups were randomly and blindly assigned into subgroups by the type of liquid ingested: either 240 ml of chocolate cow’s milk or 240 ml of a chocolate soy milk substitute, yielding four subgroups :asthmatic children + cow’s milk, asthmatic children + soy milk, non-asthmatic children + cow’s milk, and non-asthmatic children + cow’s milk. We adhered to CONSORT guidelines (http://www.consort-statement.org/) for reporting clinical trials.

Randomization took place immediately following the completion of baseline history, examination, and baseline lung function tests. It was performed by a clinical research coordinator using sequentially numbered sealed opaque envelopes containing the letter A (soy milk) or B (cow’s milk) following a randomization list generated by the website “www.randomizer.org”. This process was concealed and safeguarded by the research coordinator. The randomization list was opened by the researchers only after all participants had completed their tests. Both drinks, soy and milk, shared similar color and consistency, in efforts to assure both participant and researcher were unable to identify the intervention.

Prior to undergoing the intervention, the participants completed a questionnaire that included items pertaining to their demographic, nutritional, and medical background, the latter with specific questions regarding atopy including atopic dermatitis, allergic rhinitis and skin prick tests results for inhaled allergens. The purpose of nutritional questionnaire was to verify that all the participants consumed dairy products daily. BMI Z-scores were calculated using the Children’s Hospital of Philadelphia online Z-score calculator based on the Center for Disease Control growth charts [25]. Parents of the asthmatic children’s group completed the asthma control questionnaire (ACQ), which is a validated tool for the assessment of asthma severity [26]. All parents were asked whether they believed that milk consumption was associated with respiratory symptoms in general, and parents of the asthmatic group were also asked whether they believed that consumption of milk was associated with their child’s asthma symptoms. Clinical and pulmonary function tests were performed prior to ingestion of the assigned beverage (t_0_) and they were repeated in response to the challenge at 30, 60, 90, and 120 min post-exposure (t_30,_ t_60,_ t_90,_ t_120_, respectively). A positive response was considered as a significant change in any of these outcome measures at any time point within these 120 min.

The primary outcome of this study was a significant change in either FEV_1_ or FeNO at any time point within these 120 min compared to baseline (t_0_).

The secondary outcome measures were:

1. Any subjective clinical complaint, such as cough, phlegm, or any breathing problem or difficulty.
2. Any positive clinical findings on physical examination.
3. A significant change in oxygen saturation defined as a decrease in oxygen saturation levels > 1% from baseline.

Spirometry was performed using the Koko spirometer (nSpire Health inc. Germany) which is routinely used in our laboratory according to the acceptable standards [27]. Normal values for FeNO by the single breath technique in our laboratory are in accordance with published reference values, i.e., a cutoff of 20 parts per billion (ppb) [28]. FeNO was measured with the Niox Mino (Aerocrine, Sweden) with online recording during a single breath exhalation, according to the ERS/ATS guidelines [29]. Oxygen saturation was measured by a pulse-oximeter (Massimo Radical-7).

### Statistical analysis

The sample size was calculated for FeNO and for FEV_1_ minimal effect size (0.14), with a partial eta squared of 0.02, for 4 groups with 5 repeated measures. To achieve 80% power with an alpha of 0.05 for FeNO and FEV_1_, 22 patients in each subgroup were required. Considering a dropout of 15%, 101 patients were recruited. The statistical analysis was performed with SPSS software (IBM Corp. Released 2013. IBM SPSS Statistics for Windows, Version 22.0. Armonk, NY: IBM Corp.). The normal distribution of all continuous parameters was examined using the Kolmogorov-Smirnov test. Non-normal parameters were handled using a logarithmic transformation. Chi-squared and two-way ANOVA analyses were used to compare the demographic and baseline performances of all groups. A two-way ANCOVA with repeated measures of all spirometry parameters and FeNO was used to compare the performances of all groups and subgroups over time using the baseline measurement as a covariate. A subgroup analysis was performed for participants whose parents believed that milk consumption affected their child’s respiratory symptoms. Little’s missing completely at random (MCAR) test was performed for variables with missing values and did not show any systematic patterns in missing data (p > 0.2). There were no associations between categorical variables and missingness, including gender, asthma diagnosis, and milk treatment group. Although missingness analysis showed MCAR, we performed an additional separate analysis where median value imputation was used for patients with missing values.

## Results

Ninety-eight children were recruited into the study between June 2016 and April 2017, reaching our recruitment goal and consisting of 51 non-asthmatic children and 47 diagnosed as having asthma. Two patients, one from each group, were omitted due to inability to reliably perform pulmonary function tests. Eighty-eight children (89.8%) completed spirometry and FeNO measurements at baseline and at 30, 60, 90, and 120 minutes post-challenge, (1760 data points). Eight children missed one measurement each (3 missed 30’ FeNO, 2 missed 60’ FeNO, 2 missed FEV_1_ at 120’, and 1 missed FEV_1_ at 90’). Twenty-six of the 50 non-asthmatic patients were challenged with cow’s milk and 24 with soy substitute. Twenty-two of the 46 asthmatic children were challenged with cow’s milk and 24 with soy substitute. No unintended events were observed throughout the duration of the intervention in any of the participants.

Of the 46 asthmatic children, 23 (50%) had an ACQ score lower than 0.75, indicating well-controlled disease, while 17 (37%) had a score above 1.5, indicating poorly controlled asthma. In addition, 37 (80%) of the 46 asthmatic children reported having one or more related atopic condition: atopic dermatitis (*n* = 12), allergic rhinitis (*n* = 33), or positive skin prick tests to inhaled allergens (*n* = 29). Information on allergic status was missing for one child.

Age, gender, and BMI z-score were similar for the four subgroups. FeNO data were transformed using logarithmic transformation. The asthmatic children had significantly higher FeNO levels and lower FEV_1_, FEV_1_/FVC and FEF_25 − 75_ levels at baseline compared to the non-asthmatic controls. None of the baseline levels differed between the soy and cow’s milk intervention groups (Table 1). Oxygen saturation by pulse-oximetry was normal (≥ 97%) in all participants at baseline, again with no group or subgroup differences.

**Table 1:** Baseline characteristics

			**Asthmatic children**			**Non-asthmatic children**			***p*value (asthmatic vs. non-asthmatic) [CI]**	***p*value** **(soy vs. cow’s milk) [CI]**	***p *value (Asthmatic by Milk Interaction)**^**e**^
			**Cow’s** **Milk** **(*n*** **= 22)**	**Soy milk** **(*n*** **= 24)**	**All** **(*n*** **= 46)**	**Cow’s milk** **(*n*** **= 26)**	**Soy milk** **(*n*** **= 24)**	**All (*n*** **= 50)**			
Demographics	Age (years)		11.0 ± 3.3	10.1 ± 3.2	10.5 ± 3.2	10.4 ± 3.5	10.8 ± 2.7	10.6 ± 3.1	0.956 [-1.26-1.33]	0.702 [-1.045-1.545]	0.363
	Sex^a^		45.4	62.5	54.3	34.0	54.1	44	0.311	0.066	0.234
	BMI Z-score		0.04 ± 0.9	0.39 ± 1.4	0.23 ± 0.1.2	-0.00 ± 1.06	-0.13 ± 1.07	0.05 ±0.95	0.435 [-0.609-0.264]	0.278 [-0.675-0.196]	0.589
Parental beliefs	milk increases mucus production^b^		14 (63.6%)	12 (50%)	26 (56.5%)	6 (24%)	9 (37.5%)	15 (30.6)	0.011	0.906	0.041
	milk effects child’s respiratory symptoms^c^		5 (22.7%)	7 (29.2%)	12 (26.1%)	0	1 (4.2%)	1 (2%)	0.001 (F)	0.552 (F)	0.006
	Milk avoidance during respiratory symptoms (%)		3 (13.6%)	9 (37.5%)	12 (26.1%)	0	1 (4.2%)	(2%)	0.001 (F)	0.07 (F)	0.001
Atopy	Allergic rhinitis (%)		11 (50%)	17 (70.8%)	33 (71.7%)	2 (7.7%)	1 (4.2%)	3 (6%)	< 0.0001 (F)	1	< 0.0001
	Positive skin prick tests to inhaled allergens (%)		14 (63.6%)	15 (62.5%)	29 (63%)	3 (11.5%)	0	3 (6%)	< 0.0001 (F)	0.665	< 0.0001
	Atopic dermatitis (%)		5 (22.7%)	7 (29.2%)	12 (26.1%)	1 (3.8%)	1 (4.2%)	2 (4%)	0.003 (F)	0.563	0.021
	ACQ scores		0.930 ± 1.033	1.144 ± 1.116	1.032 ± 1,067					0.504	
Baseline spirometry and FeNO	FEV_1_ t_0_^d^		90.5 ± 17.0	96.8 ± 13.7	93.8 ± 15.5	101.0 ± 12.8	100.8 ± 11.3	100.9 ± 11.7	0.013 [1.52–12.60]	0.353 [-8.4-3.0]	0.248
	FEV/_1_FVC t_0_^d^		95.8 ± 10.3	94.1 ± 21.5	94.9 ± 16.9	103.3 ± 7.2	104 ± 5.4	103.6 ± 6.4	< 0.01 [3.36–13.97]	0.764 [-4.57-6.20]	0.646
	FEF25-75 t_0_^d^		80.3 ± 22.6	83 ± 28.3	81.7 ± 25.5	100.2 ± 22.9	99.6 ± 18.8	99.9 ± 20.8	< 0.01 [8.82–27.62]	0.967 [-10.31-9.89]	0.724
	FeNO (ppb) t_0_		40.6 ± 37.6	45.6 ± 41.0	43 ± 38.9	13.2 ± 13.0	14.7 ± 7.3	13.9 ± 10.4			
	Log FeNO		3.43 ± 0.92	3.34 ± 0.86	3.39 ± 0.88	2.57 ± 0.49	2.33 ± 0.66	2.45 ± 0.59	< 0.0001 [-1.251-0.626]	0.314 [-0.544-0.176]	0.859

Values are given mean ± SD

^a^Percentage of males, Chi-square

^b^Percentage of parents believing milk increases mucus production

^c^Percentage of parents believing milk effects their child’s respiratory symptoms

^d^Percent predicted

^e^Two-way ANOVA for Asthmatic GroupXMilk Group interaction

**Fig. 1:**
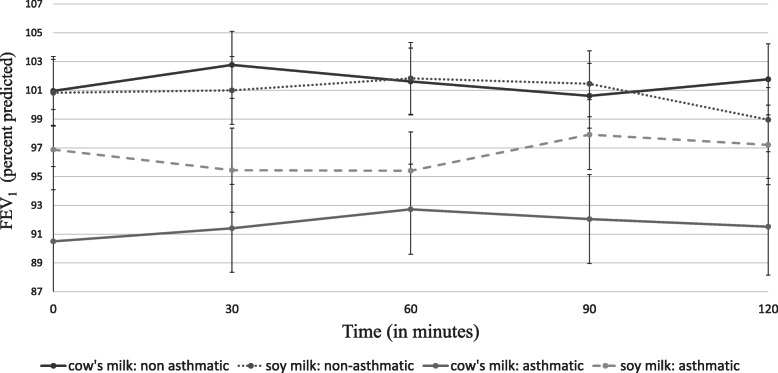
Changes in forced expiratory volume in 1 s (FEV_1_) over time by subgroups. Legend: Values are mean ± standard error. There was no difference between cow’s milk and soy milk for any of the time points (*n* = 91, *p =* 0.395) or between any of those time points for the asthmatic and non-asthmatic groups (*p* = 0.224)

**Fig. 2:**
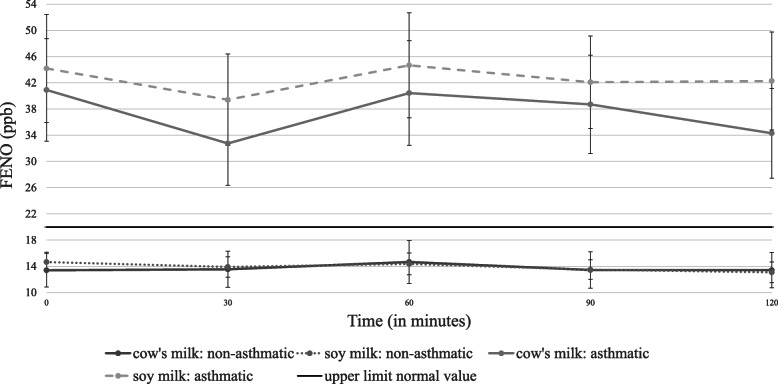
Changes in fractional exhaled nitric oxide (FeNO) over time. Legend: Values are mean ± standard error. There was no difference between cow’s milk and soy milk for any of the time points (*n* = 88, *p* = 0.641), or between any of those time points for the asthmatic and non-asthmatic groups (*p* = 0.529)

**Fig. 3:**
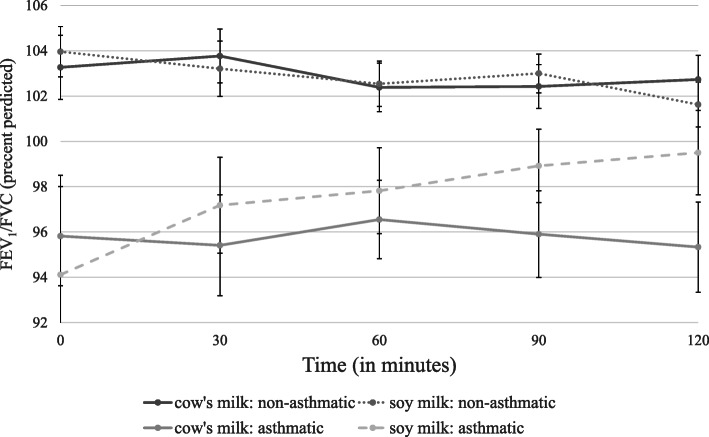
Changes in FEV_1_/FVC over time. Legend: Values are mean ± standard error. There was no difference between cow’s milk and soy milk for any of the time points (*n* = 91, *p* = 0.735), or between any of those time points for the asthmatic and non-asthmatic groups (*p* = 0.307)

No change in clinical findings was observed among any of the participants at any time point following the cow’s milk or soy challenges compared to baseline (Table 2). There was no change in oxygen saturation. Due to baseline differences in FeNO and spirometry parameters, repeated measures ANCOVA were controlled for the baseline parameters values. There was no significant asthma by time interactions in all parameters (Table 3). There was no interaction between asthmatic and non-asthmatics groups, the intervention (milk vs. soy) groups and FEV_1_ over time. Similarly, no interaction was found between asthmatic and non-asthmatics groups, the intervention groups and FeNO over time (Table 3; Figs. 1, 2 and 3). Missing values imputation using median values had no effect on repeated measures analysis.

**Table 2:** Spirometry and FeNO means before and following intervention

		Asthmatic children		Healthy children	
		**Cow’s milk (*n* = 22)**	**Soy milk (*n* = 24)**	**Cow’s milk (*n* = 26)**	**Soy milk (*n* = 24)**
FEV_1_ mean [95% CI]	**Baseline**	90.5 [50.0-112.4]	96.8 [73.2-123.5]	101.0 [76.1–121]	100.8 [82.8-126.5]
	**30 min**	91.4 [58.6-117.5]	95.5 [70.2-122.7]	102.8 [84.5-122.7]	101.0 [83.0-129.0]
	**60 min**	92.7 [58.6-114.8]	95.4 [74.4-120.7]	101.6 [82.7-122.7]	101.8 [82.5-131.5]
	**90 min**	92.1 [58.3-110.85]	97.9 [79.2–124.0]	100.6 [84.4-124.6]	101.5 [84.0-129.0]
	**120 min**	91.5 [60.2-118.3]	97.2 [78.2-123.3]	101.8 [83.0-123.7]	99.0 [74.3-120.3]
FEV_1_/FVC mean [95% CI]	**Baseline**	95.8 [71.6-114.4]	94.1 [79.0-110.0]	103.3 [82.9-114.6]	104 [92.5-114.8]
	**30 min**	95.4 [70.6-115.7]	97.2 [76.4-114.4]	103.8 [88.8-113.3]	103.2 [93.5-114.5]
	**60 min**	96.6 [81.1-111.7]	97.8 [79.6-111.6]	102.4 [87.7-110.7]	102.5 [93.3–110.0]
	**90 min**	95.9 [75.5-111.7]	98.9 [78.8-108.8]	102.4 [93.4-114.9]	103.0 [95.3-111.8]
	**120 min**	95.3 [75.8-111.9]	99.5 [79.5-113.5]	102.7 [89.5-111.7]	101.6 [92.3-110.5]
FeNO mean [95% CI]	**Baseline**	40.6 [8.1-123.4]	45.6 [5.1-169.4]	13.2 [5.0-54.1]	14.7 [4.8–32.7]
	**30 min**	32.8 [4.1-104.1]	39.4 [5.0-126.2]	13.5 [5.0-59.1]	13.9 [5.2–32.0]
	**60 min**	40.4 [8.1-125.1]	44.7 [6.0-161.9]	14.7 [5.2–69.2]	14.4 [6.0–33.0]
	**90 min**	38.7 [6.3-111.4]	42.1 [5.1-138.4]	13.4 [5.0-58.7]	13.5 [5.2–31.7]
	**120 min**	34.3 [7.1–98.6]	42.3 [5.0-151.8]	13.4 [4.4–56.6]	13.1 [4.7–32.5]

**Table 3:** Repeated measures ANCOVA analysis of spirometry and FeNO parameters (*p*-values)

	*Asthma Main effect (between groups)*	*Milk Main effect (between groups)*	*Asthma by Milk Main effect (between groups)*	*Interaction Asthma-by-time (within groups)*	*Asthma-by-Milk-by time interaction (within groups)*
**FeNO**	0.529	0.641	0.213	0.855	0.982
**FEV**_**1**_**(predicted)**	0.224	0.395	0.997	0.111	0.446
**FEV**_**1**_**/FVC**	0.307	0.735	0.214	0.217	0.183
**FEF25-75**	0.334	0.625	0.243	0.160	0.309

Parents of 12/46 asthmatic children reported that they believed that milk consumption affected respiratory symptoms in their child, including onetime milk consumption. A subgroup analysis revealed no difference between the 12 asthmatic participants whose parents held this belief and the asthmatic participants whose parents did not share this perception.

## Discussion

The results of our study did not demonstrate any association between the cow milk challenge and acute bronchoconstriction or eosinophilic inflammation in either non-asthmatic or asthmatic children. We used soy milk as a placebo to control for any subjective or physiologic effect of the procedures themselves.

Our results, showing that acute exposure to cow’s milk did not affect symptoms, airway physiology, or FeNO in healthy children, were expected. Nevertheless, our finding that acute exposure to cow milk did not affect these variables also in asthmatic children is of major importance, since this is the group in which the avoidance of dairy products is more common and “rationalized” by their diagnosis of asthma [8, 9]. Undoubtedly, there are other common “health justifications” for dairy avoidance, such as aggravation of atopic dermatitis [30] or gastrointestinal symptoms due to lactose intolerance [31].

Our findings correlate with the observations on these issues in adults. One study from Australia reported that participants who believed in the relationship between cow’s milk and mucus production were more likely to report sensations related to difficulty in swallowing and to perceived thickness of mucus and salivary secretions compared to non-believers [5]. Notably, these subjective sensations occurred regardless of whether they drank cow’s milk or soy milk, with no significant difference between them. In another report from Australia, Pinnock et al. studied respiratory symptoms and consumption of cow’s milk in 60 adults who volunteered to be infected with rhinovirus, and found no association between milk consumption and those symptoms [10]. A third Australian study compared exposure to cow’s milk among 10 asthmatic adults who reported exacerbations following exposure to milk products with 10 controls who consumed rice milk. No association was observed between milk ingestion and respiratory functions regardless of the participant’s prior perceptions [11]. Nguyen also reported not having observed any acute or delayed deterioration of pulmonary functions among 25 atopic asthmatic adults exposed to cow’s milk versus placebo [32]. Of note, asthmatic patients with known milk allergy act differently. Eighty-six percent of asthmatic adults with bronchial asthma and p ositive skin tests for milk developed a positive asthmatic response to milk challenge measured by spirometry [33]. Nevertheless, our study excluded children with present known allergy to cow’s milk.

The importance of our findings stems from the widespread belief that cow’s milk consumption provokes respiratory symptoms. This study aims to provide clinicians with evidence to convince parents who eliminate or consider eliminating dairy products from their children’s’ diet that avoiding dairy for respiratory concerns will not provide any protective effect, and might even lead to harmful effects by denying them the well-documented benefits of cow’s milk consumption in childhood and early adulthood.

This study has several limitations. First, we explored the effect of a single exposure of cow’s milk on respiratory parameters. It is still plausible that continuous exposure over days or weeks will result in decreased spirometric values and increased FeNO. Even though none of our participants displayed any symptoms or significant change in lung function tests, a more substantial exposure would help strengthen our findings. Furthermore, one might argue that the pathogenesis underlying increased mucus production following milk exposure is delayed-type hypersensitivity. This type of hypersensitivity may take 2 or more days to develop [34], thus an endpoint of two hours following intervention will not be sufficient to detect any change from baseline values. Nevertheless, none of the parent reported the occurrence of any symptoms in the days following the study. Second, our participants, who consume dairy products regularly, eliminated them for 24 h prior to the exposure. Neither the inclusion of dairy products in one’s regular diet nor their elimination during a given period should be expected to affect healthy children, since their baseline clinical and laboratory status were normal and thus no improvement in respiratory parameters would be expected after a longer elimination time. Nevertheless, we cannot exclude the possibility that a longer elimination of dairy products would result in higher baseline FEV_1_ and lower FeNO levels in asthmatics.

On recruitment, we evaluated patients’ health comprising present and past history including allergies, nevertheless, we did not explicitly ask regarding past and resolved milk allergy or intolerance, hence, it is possible that such participants were included. However, since none of the participants showed a positive response to the challenge, such a situation did not affect the results. Whether a positive history can affect the response to challenge should be investigated in a different study.

Finally, while our study did not show any association between exposure to cow’s milk and respiratory symptoms or abnormal pulmonary functions among the patients whose parents believed that milk was associated their children’s’ respiratory symptoms, that subgroup consisted of only 12 participants.

## Conclusions

Acute exposure to cow’s milk is not associated with short-term respiratory symptoms, airway inflammation, or bronchial constriction in both non-asthmatic and asthmatic children. The elimination of cow’s milk from a child’s diet for respiratory considerations is not supported by the evidence that emerged from this study. Further studies for exploring longer elimination periods and prolonged exposure are warranted.

## Data Availability

All data generated or analyzed during the current study are available from the corresponding author on reasonable request.

## Abbreviations

BMI: Body Mass Index
FeNO: Fractional exhaled Nitric Oxide
FEV_1_: Forced Expiratory Volume during the first second
FVC: Forced Vital Capacity
FEF_25-75_: Forced Expiratory Flow at 25-75%
PPB: Parts per Billion
MCAR: Missing Completely at Random
CI: Confidence interval
